# Stripping Away the Soil: Plant Growth Promoting Microbiology Opportunities in Aquaponics

**DOI:** 10.3389/fmicb.2018.00008

**Published:** 2018-01-22

**Authors:** Ryan P. Bartelme, Ben O. Oyserman, Jesse E. Blom, Osvaldo J. Sepulveda-Villet, Ryan J. Newton

**Affiliations:** ^1^School of Freshwater Sciences, University of Wisconsin-Milwaukee, Milwaukee, WI, United States; ^2^Bioinformatics Group, Wageningen University & Research, Wageningen, Netherlands; ^3^Department of Microbial Ecology, Netherlands Institute of Ecology, Wageningen, Netherlands; ^4^Johns Hopkins Center for a Livable Future, Department of Environmental Health and Engineering, Bloomberg School of Public Health, Johns Hopkins University, Baltimore, MD, United States

**Keywords:** aquaponics, plant growth promoting microorganisms, recirculating aquaculture, chlorosis, rhizosphere, microbiome

## Abstract

As the processes facilitated by plant growth promoting microorganisms (PGPMs) become better characterized, it is evident that PGPMs may be critical for successful sustainable agricultural practices. Microbes enrich plant growth through various mechanisms, such as enhancing resistance to disease and drought, producing beneficial molecules, and supplying nutrients and trace metals to the plant rhizosphere. Previous studies of PGPMs have focused primarily on soil-based crops. In contrast, aquaponics is a water-based agricultural system, in which production relies upon internal nutrient recycling to co-cultivate plants with fish. This arrangement has management benefits compared to soil-based agriculture, as system components may be designed to directly harness microbial processes that make nutrients bioavailable to plants in downstream components. However, aquaponic systems also present unique management challenges. Microbes may compete with plants for certain micronutrients, such as iron, which makes exogenous supplementation necessary, adding production cost and process complexity, and limiting profitability and system sustainability. Research on PGPMs in aquaponic systems currently lags behind traditional agricultural systems, however, it is clear that certain parallels in nutrient use and plant-microbe interactions are retained from soil-based agricultural systems.

## Aquaponics – Stripping Away the Soil

Aquaponics, the combined culture of fish and plants in recirculating water systems was pioneered in the 1970s (Sneed et al., 1975; Naegel, 1977; Lewis et al., 1978) as an environmentally sustainable agricultural method based on the concepts of minimal water use and minimal impact on environmental water quality compared to traditional agricultural methods (Blidariu and Grozea, 2011). In addition to producing salable crops, aquaponics is valued for its positive development of community and economic opportunity in urban areas (Goodman, 2011), and its wide-ranging educational benefits for students through the post-secondary level (Hart et al., 2014; Genello et al., 2015). Despite these benefits, microbial research supporting aquaponic crop production lags behind traditional agricultural systems. Here, we present the case that aquaponic systems provide relatively untapped potential for research on plant-microbe interactions.

Aquaponics is a highly engineered agricultural system that uses fish effluent (which comprises both particulate waste solids and dissolved nutrients) from a recirculating aquaculture subsystem as nutrient medium to grow edible plants in attached hydroponic subsystems. In return, nutrient removal through plant absorption and growth, parallel to their associated microbiota, decreases dissolved solid and ionic concentrations, which, in turn, benefits fish production by improving overall water quality parameters, including the removal of toxic metabolites, such as ammonia and nitrite. Together, this circular and beneficial relationship between fish, plants, and microbes reduces water usage compared to traditional agriculture. Historically, aquaponics research was driven by the recirculating aquaculture community; so most technological advancement was focused on optimizing water quality for fish production purposes (Sneed et al., 1975; Naegel, 1977; Lewis et al., 1978). However, recent energy analyses and industry surveys of aquaponic systems concluded that profitability is greater when a plant-centric production approach is adopted (Love et al., 2015a,b). Incorporation and testing of hydroponic method compatibility is therefore critical to integrating simultaneous fish and plant production. For example, using a hydroponic-centered production setup, Schmautz et al. (2016) found that the aquaculture-subsystem provided the necessary nutrients to produce cherry tomatoes with nutrient content that exceeded those available at local markets. However, to improve plant growth in plant-centric aquaponic systems, micronutrient supplementation (e.g., iron, calcium, and potassium) is often required (Rakocy et al., 2004; Roosta, 2014; Bittsanszky et al., 2016). Additionally, chemical inputs are needed to counteract disease and other plant stressors (Nguyen et al., 2016), which adds long-term operational expenses (Love et al., 2015a). Thus, despite the benefits of these integrated agricultural systems, high capital expenses during system construction (Engle, 2015), and the abovementioned chemical supplementation make achieving economic sustainability a challenge. Continued research on micronutrient transport and transformation between aquaponic subsystems is needed to identify shortcomings and optimize engineering paradigms in aquaponic systems.

Plant growth promoting microorganisms (PGPMs) may be an effective alternative to chemical inputs for dealing with plant growth requirements and stressors in aquaponic systems. Plants recruit PGPMs from the surrounding environment to their rhizosphere using specific chemical signaling (DeVries and Wallenstein, 2017). For example, under phosphorus (P)-limiting conditions, the plant hormone strigolactone is released into the rhizosphere, where it serves as a signaling molecule to initiate associations with fungi (Akiyama et al., 2005). In soil-based environments PGPMs are known to enhance plant growth via a number of mechanisms, including: nitrogen fixation, organic matter mineralization, root growth promotion, protection against pathogens, and increasing the bioavailability of nutrients, including micronutrients such as iron (Höflich et al., 1995; Loper and Henkels, 1997, 1999; Mendes et al., 2011; Loper et al., 2012; Malusá et al., 2012; Marasco et al., 2012; Rashid et al., 2012; Coleman-Derr and Tringe, 2014; Dias et al., 2014; Pii et al., 2015; da Silva Cerozi and Fitzsimmons, 2016; Khalifa et al., 2016). In soilless environments, PGPM research is limited, but existing studies suggest PGPMs also play a significant role in plant growth and health (Gravel et al., 2006; Villarroel et al., 2011; da Silva Cerozi and Fitzsimmons, 2016; Nguyen et al., 2016; Sheridan et al., 2016).

Regardless of the agricultural system, root health is essential to the survival of plants; so one focus area for aquaponic PGPM research should be microbial root colonization. Arbuscular mycorrhizal fungi are well-documented plant growth promoting fungi that colonize plant roots. In traditional soil-based agriculture, arbuscular mycorrhizal fungi promote phosphorus uptake and enhance biomass production (Govindasamy et al., 2011). Arbuscular mycorrhizal fungi also appear to be important for plant health in hydroponics. For example, in one hydroponic system study, arbuscular mycorrhizal fungi inhibited *Fusarium* spp. from inducing root rot in tomatoes grown under near-commercial conditions (Utkhede, 2006). While arbuscular mycorrhizal fungi are a commonly cited PGPM, many different microorganisms are thought to be PGPMs. One such group, rhizobia, were discovered in the 19th Century (Beijerinck, 1888), and now these diazotrophic bacteria are recognized as essential agents in promoting growth among crops such as legumes, rice, and wheat (Govindasamy et al., 2011; Ji et al., 2014; Majeed et al., 2015). Interestingly, iron siderophores facilitate the formation of rhizobium diazotrophic nodules (Barton et al., 1996; Brear et al., 2013), suggesting micronutrients may play a role in PGPM colonization in other agricultural systems, such as hydroponics/aquaponics. However, some PGPM benefits may come at an adaptive cost, such as increased sensitivity to insect herbivory (Barazani and Baldwin, 2013), but controlled environmental agriculture can account for invertebrate pest problems (Fox et al., 2012). Ultimately, research on PGPM in aquaponic systems may alleviate costly nutrient supplementation by properly integrating PGPM driven environmental processes into system design.

In addition to benefitting aquaponic crop production, PGPM research in soilless engineered environments has the potential to advance the fundamental understanding of rhizosphere microorganism associations. It is clear plants recruit PGPMs to their rhizosphere, but the mechanisms driving plant growth promoting rhizosphere interactions are difficult to disinter from soil-based studies (DeVries and Wallenstein, 2017). Soil matrices are chemically complex and heterogeneous, exhibiting immensely diverse microbial communities. Additionally, given the large variability among soil and crop types (DeVries and Wallenstein, 2017), rhizosphere recruitment of PGPMs in this environment remains mainly theoretical and often limited to a case-by-case basis. The complexity of the soil matrix also adds technological hurdles to studying PGPMs. The soil matrix often hinders nucleic acid extraction and, subsequently, sequence-based analyses of microorganisms, thus inhibiting the exploration of microbial community structure (Carini et al., 2016), genetic signatures pertaining to nutrient processing (Krsek and Wellington, 1999; Martin-Laurent et al., 2001), and the identification of microbial guilds to utilize in rhizosphere engineering (Savka et al., 2013; Mueller and Sachs, 2015; Pii et al., 2015; Dessaux et al., 2016). In contrast to soil-based agricultural systems, aquaponic systems operate in highly monitored and controlled environments (e.g., pH, temperature, hydraulic retention time, nutrient concentrations, etc.), and lack the confounding variability and complexity of the soil-matrix. As a result, these systems represent, scalable, highly reproducible, and adjustable laboratories for PGPM research, where discoveries made in a research setting may be more directly transferred to an industrial or practical application.

## The Challenges of Integrated System Design in Aquaponics

Aquaponic systems must balance the physiological requirements of both plant and fish in order to maintain their health. This balance makes even basic system design challenging, such as identifying plant and fish species that are compatible. The integration of distinct fish and plant subsystems also means operational change in any one component inherently impacts all other components, thereby creating a fairly high level of ecological complexity. For example, solid waste in aquaponic systems primarily consists of fish feed and feces, which, when decomposed, act as fertilizer for the hydroponic subsystem. Calculated fish feed rates relate to plant grow-bed size, but feed conversion and nutrient assimilation varies with feed protein type (i.e., plant-derived vs. teleost protein extracts) and plant crop-type (Rakocy et al., 2006; Timmons and Ebeling, 2013; Hu et al., 2015; Medina et al., 2016). Excess solid waste increases oxygen demand leading to hypoxic conditions in the rhizosphere, and may generate toxic concentrations of ammonia and nitrite (Rakocy, 2012; Danaher et al., 2013). Therefore, proper solids management is necessary to maintain the oxygen gradient around the plant roots allowing for colonization of PGPMs and preventing phytopathogen growth. However, in the roots of the hydroponic subsystem an appropriate level of solid waste re-mineralization is essential to supply micro- and macronutrients to the plants (Rakocy et al., 2012). Basic system design constraints influence overall microbial community structure and suggest microbial niche differentiation within system components, but the influence of environmental conditions has yet to be explored in aquaponic systems (Schmautz et al., 2017). Food web interactions, including predation on bacteria and archaea and nutrient or energy transfer from microbial eukaryotic activity, such as that from micro-fungi, may also confound system designs in yet unknown ways. These food web interactions have had little consideration in system designs to date, but deserve thorough analysis as control points for microbial–plant interactions and in experiments aimed at optimizing aquaponic system technology.

## Iron Limitation: a Case Study for Pgpm Research in Aquaponic Plant Production

Commonly, iron is supplemented in the hydroponic subsystems of aquaponics configurations; however, little attention has been paid to exactly why this supplementation is required. Herein we review what is known about iron requirements in aquaponics and discuss possible iron supplementation strategies that do not require industrially produced chelated iron. Iron is an essential molecule for a multitude of metalloprotein structures (e.g., hemoglobin, chlorophyll, and cytochromes), and therefore, demand is high from all biological components of an aquaponic system. Fish assimilate low amounts of iron relative to terrestrial livestock (van Dijk et al., 1975), and often fish iron needs are met or frequently exceeded, with commercial feeds (Watanabe et al., 1997), so little attention is paid to this component of aquaculture operations. In contrast, although undigested fish feed contains excess iron, plant grow beds in aquaponics are often limited by bioavailable iron (Fe^2+^). This nutrient deficiency is a known cause of chlorosis in the hydroponic subsystem crops, but may not be evident in a system until vegetable products have been raised for multiple generations (Rakocy et al., 2004). In the University of the Virgin Islands system, chelated iron is added at a concentration of 2 mg/L per day to prevent chlorosis (Rakocy et al., 2004). One major factor driving iron deficiency is that soluble ferrous iron (2+) easily crosses the rhizoplane of the roots, but ferric iron (3+) is insoluble. Consequently, the competing chemical reactions driving Fe^2+^ to Fe^3+^ (i.e., the speciation of iron in natural water systems by hydroxyl radicals and ionic interactions) and pH dependency complicate the mass balance of iron in aquaponic systems (Rose and Waite, 2002; Waite, 2002). Biotic factors in aquaponic systems may also reduce available iron for plants. Endemic microbial communities scavenge iron for constructing metalloprotein centers (Andrews et al., 2003), but in aquaponics the extent of this iron demand remains unknown.

Limitation of biologically available iron is a relatively common phenomenon across environments containing photosynthetic organisms. One such environment is the ocean, where primary productivity depends on soluble iron, though iron remains sequestered in microbial amphiphilic siderophores (Boiteau et al., 2016). Published literature on the role of siderophores in soil-based agriculture point to both an enhancement of growth and a link to pathogenesis (Kloepper et al., 1980; Neilands and Leong, 1986). *Pseudomonas fluorescens* Pf-5 is one PGPM known to increase the bioavailability of iron through siderophore production in iron deficient soils (Loper and Henkels, 1997). Interestingly, some pseudomonads, like *Pseudomonas putida*, are able to scavenge iron from other siderophores under laboratory conditions and promote iron uptake in experimental cucumber seedlings (Loper and Henkels, 1997). The “siderophore theft” may be indicative of pseudomonad PGPM’s ability to remediate disease (Loper and Buyer, 1991; Loper and Henkels, 1997). Furthermore, genomic analyses of *Pseudomonas* spp. indicate a distinct ability to modulate the surrounding rhizosphere community through antifungal and bacteriocin production, in addition to siderophore production (Loper et al., 2012). In soil-based agriculture, other bacterial species such as, *Bacillus* and *Paenibacillus* spp. exhibit similar PGPM characteristics (Govindasamy et al., 2011). All of the aforementioned PGPM microbes found in soil studies may allow aquaponics practitioners to biologically remediate regularly occurring nutrient deficiencies. Since there is evidence PGPM ecophysiologies work to overcome micronutrient deficiencies in engineered environments (Villarroel et al., 2011), there is ample opportunity to research their usefulness for aquaponic system design and management.

All nutrient rich hydroponic systems, including aquaponics, must balance the promotion of beneficial microorganisms while minimizing the growth and rapid spread of plant pathogens (Lee and Lee, 2015). Though the resiliency of aquaponic systems to phytopathogen infection requires experimental study, other groups of PGPM’s (such as *Bacillus* spp. and *Paenibacillus* spp.) may be linked to plant resilience (Govindasamy et al., 2011; Loper et al., 2012). In hydroponics, PGPM species have been identified from the bacterial genera *Pseudomonas, Bacillus, Enterobacter, Streptomyces, Gliocladium*, and *Trichoderma*; many of which produce siderophores (Lee and Lee, 2015). It also has been demonstrated that siderophore production by a *Chryseobacterium* spp. alleviates iron starvation in tomato plants (Radzki et al., 2013). It is likely that other syntrophic or symbiotic relationships between plants and rhizoplane microbiomes exist, but as of now remain undiscovered or underutilized. As more PGPM’s are discovered, operators may potentially integrate batch-culturing devices to facilitate the growth of PGPM’s producing siderophores. Batch production could minimize industrially manufactured chelated iron input into the system, while aiding producers in ending the dispute over USDA organic certification for aquaponic and hydroponic produce (Biernbaum et al., 2016). At the 2017 National Organic Standards Board meeting no decision was made as to whether aquaponic or hydroponic crops could be certified organic under United States law.

Microbial iron use is a complicating factor in all environments with photosynthetic activity. In aquaponic systems, iron demand upstream of the hydroponic subsystem could induce a nutrient sink unless microbial micronutrient acquisition is considered in aquaponic system engineering. For example, an important design feature of aquaponics and recirculating aquaculture is solid waste decomposition, which induces anoxic or hypoxic conditions and facilitates methanogen growth when solid retention times (SRTs) are greater than 10 days (Suhr et al., 2015). System iron deficits may be compounded if solids management component SRT allows for methanogenic growth, since many methanogens and methanotrophs require iron in metallo-protein complexes to catalyze reactions (Speece, 1983; Glass and Orphan, 2012; Ettwig et al., 2016). Additionally, iron is closely linked to the nitrogen cycle; a critical nutrient cycle to manage for both fish and plant growth (Klotz and Stein, 2008; Timmons and Ebeling, 2013; Glass, 2015). In some systems, such as the ocean or recirculating aquaculture system solids digesters, the link between iron and nitrogen is significant, as denitrification pathways found in heterotrophic microorganisms constitute one of the largest group of iron dependent metabolic pathways (van Rijn et al., 2006; Glass et al., 2015). Careful consideration of solids management and microbial nitrification is essential to iron management in aquaponics, if siderophore-producing PGPM are to successfully mitigate chlorosis.

## The Microbial Future of Aquaponics

Integrating PGPMs into aquaponic system design has the potential to alleviate micronutrient fluctuations and phytopathogen blooms in the hydroponic subsystem of an aquaponic system. PGPM use in aquaponic process engineering may maintain optimal plant production with lower nutrient concentrations than those found in a typical commercial hydroponic system, thereby reducing the incidence of disease, and abiotic inhibition of plant nutrient uptake (Mill et al., 1996; Rakocy et al., 1997; Lee and Lee, 2015). These operational conditions also may be maintained in a hydroponic system, but without fish feed and feces serving as the basis of plant growth substrate, external costs and maintenance increase (Villarroel et al., 2011). Typically, the aquaponic system operator supplements iron, calcium, and potassium, but solid waste re-mineralization reduces supplementation cost compared to stand alone hydroponic systems (Rakocy et al., 2006; Bittsanszky et al., 2016). Raising Tilapia (*Oreochromis niloticus* L.) at low stocking densities was found to reduce nutrient costs incurred in hydroponic strawberry production, however, PGPM growth was not considered (Villarroel et al., 2011). These results and those from other studies (e.g., Schmautz et al., 2016), suggest that fish effluent could serve as primary growth media for hydroponic subsystems, but supplemental nutrients may be needed depending on the stocking density of the fish, plant crop grown, and presence of active PGPM.

Besides PGPM research, there are a number of additional areas where microbial-based research could benefit aquaponic production. For example, aquaponic practitioners must carefully balance the pH requirements of fish, nitrifying microorganisms, and plants by identifying a mean pH that facilitates biological growth throughout all components, even if it is not optimal for any one component. Typically this means aquaponic operation at a pH of 7.0, whereas plants grown hydroponically prefer a lower pH, from 5.5 to 6.5 (Rakocy et al., 2006). However, pH balancing does not follow a concrete rule for operation, as a review of aquaponic crop production (Tyson et al., 2011) suggests normal total crop yields may be obtained at pH levels above those recommended for traditional production. Recent research into nitrifying microorganisms suggests that certain species of nitrite-oxidizing bacteria become more competitive at lower pH levels (Hüpeden et al., 2016), presenting the opportunity for further research into operating aquaponic systems at a lower pH and allowing pH to be optimized for specific plant or PGPM growth. There is also evidence of overlap between fish gut microbiomes and rhizosphere microbiomes (Hacquard et al., 2015), which could indicate microbial-based health benefits of fish-plant co-cultivation and an opportunity to use PGPM manipulation to benefit both plant and fish growth. Root-associated microorganisms are also known to influence plant phenology, such as flowering time (Wagner et al., 2014; Pérez-Jaramillo et al., 2016), and thus, in theory, could be used to manipulate desired plant biological characteristics in controlled settings such as those found in aquaponics. Finally, a recent model argues that a shift from continuous recirculation to decoupling aquaponic system components could lower incidences of nutrient supplementation and allow for PGPM growth planned into initial aquaponic system design (Goddek et al., 2016). Decoupling aquaponic components could free greater quantities of allochthonous iron and micronutrients from solid waste, while also sustaining crop growth during emergency or routine maintenance, but further research in this area and all those mentioned above is needed.

Despite the benefits of these integrated agricultural systems, achieving economic sustainability of aquaponic systems remains a challenge, primarily due to high capital expenses during system construction (Engle, 2015) and the need to reduce recurrent operational expenses (Love et al., 2015a). Microbial ecological theories governing nutrient cycling, host interactions, and community assembly underpin plant growth and health in aquaponic/hydroponic systems (Blancheton et al., 2013). Testing, understanding, and applying these theories to improve system design is crucial as global protein demands are becoming ever more reliant upon aquaculture/aquaponic systems, and many are turning to aquaponics to serve food deserts in cities and decrease agricultural water usage (Blidariu and Grozea, 2011; Goodman, 2011; FAO, 2014). We advocate that one “moon shot” research area for microbiologists should be enhancing sustainable agricultural systems, as this area presents opportunities to decentralize the food production system, simplifies agricultural logistics by decreasing distance to market, and enables more food safety and quality controls than traditional agriculture, while positively impacting local economies.

## Author Contributions

RB and JB developed and outlined the initial concept for the perspective. RB was the primary author and lead on writing and editing. BO, JB, and OS-V contributed ideas and writing for particular sections relating to their expertise, and all were involved in writing and editing of manuscript drafts. RN contributed to the initial outline and perspective focus, was involved in writing and editing of all manuscript stages, and was the primary source of funding for the project.

## Conflict of Interest Statement

The authors declare that the research was conducted in the absence of any commercial or financial relationships that could be construed as a potential conflict of interest.
